# Controlling accumulation of fermentation inhibitors in biorefinery recycle water using microbial fuel cells

**DOI:** 10.1186/1754-6834-2-7

**Published:** 2009-04-01

**Authors:** Abhijeet P Borole, Jonathan R Mielenz, Tatiana A Vishnivetskaya, Choo Y Hamilton

**Affiliations:** 1 BioSciences Division, Oak Ridge National Laboratory, Oak Ridge, TN 37831-6226, USA; 2 The University of Tennessee, Knoxville, TN 37996, USA

## Abstract

**Background:**

Microbial fuel cells (MFC) and microbial electrolysis cells are electrical devices that treat water using microorganisms and convert soluble organic matter into electricity and hydrogen, respectively. Emerging cellulosic biorefineries are expected to use large amounts of water during production of ethanol. Pretreatment of cellulosic biomass results in production of fermentation inhibitors which accumulate in process water and make the water recycle process difficult. Use of MFCs to remove the inhibitory sugar and lignin degradation products from recycle water is investigated in this study.

**Results:**

Use of an MFC to reduce the levels of furfural, 5-hydroxymethylfurfural, vanillic acid, 4-hydroxybenzaldehyde and 4-hydroxyacetophenone while simultaneously producing electricity is demonstrated here. An integrated MFC design approach was used which resulted in high power densities for the MFC, reaching up to 3700 mW/m^2 ^(356 W/m^3 ^net anode volume) and a coulombic efficiency of 69%. The exoelectrogenic microbial consortium enriched in the anode was characterized using a 16S rRNA clone library method. A unique exoelectrogenic microbial consortium dominated by δ-Proteobacteria (50%), along with β-Proteobacteria (28%), α-Proteobacteria (14%), γ-Proteobacteria (6%) and others was identified. The consortium demonstrated broad substrate specificity, ability to handle high inhibitor concentrations (5 to 20 mM) with near complete removal, while maintaining long-term stability with respect to power production.

**Conclusion:**

Use of MFCs for removing fermentation inhibitors has implications for: 1) enabling higher ethanol yields at high biomass loading in cellulosic ethanol biorefineries, 2) improved water recycle and 3) electricity production up to 25% of total biorefinery power needs.

## Background

**Figure 1:**
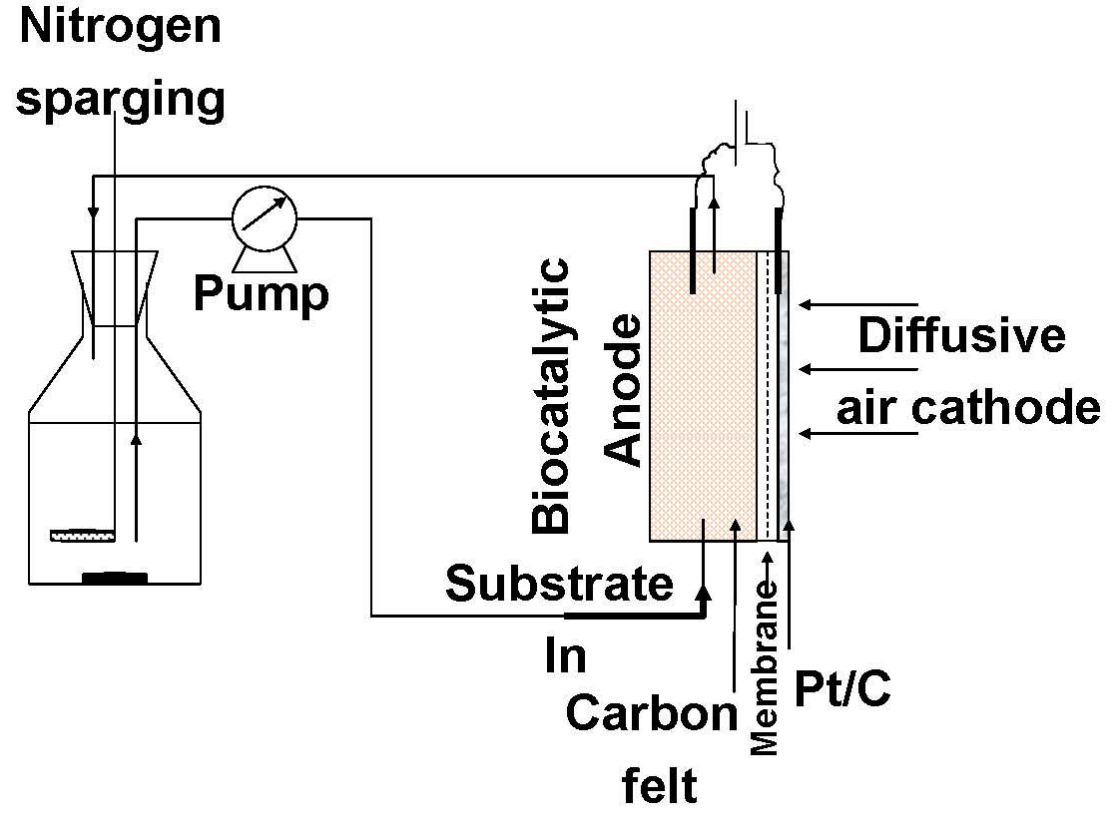
**Representation of a biofuel cell with a microbial anode**.

Microbial fuel cells (MFCs) are devices which convert organic matter to energy (electricity or hydrogen) using microorganisms as catalysts (Figure 1). Conversion of sugars, organic acids and other degradable matter to electricity has been demonstrated [1,2]. The use of this technology for electricity production is currently limited by power density [3] and bioelectrochemical losses [4], although significant effort is being made to overcome these limitations [5-12]. The primary targets for application of this technology include wastewater treatment with simultaneous electricity production and low-power-utilizing remote sensors, although many other applications are being investigated [5,13,14]. Significant progress has been made in recent years in understanding the factors affecting the power density. We have recently reported developing integrated anode designs and biocatalyst enrichments by combining multiple modes resulting in power densities above 300 W/m^3 ^[15], which is approaching the power densities needed for commercial consideration [4]. Improvements in developing sustainable cathode design can bring this technology closer to commercialization [16-18]. Additionally, production of hydrogen instead of electricity can significantly improve economics [19-21].

Biochemical conversion of renewable feedstocks to ethanol is being investigated at commercial scale for biofuels production [22,23]. Processing biomass for biochemical conversion of polymeric carbohydrates by fermentation requires an initial thermochemical step, called pretreatment, using either acidic or basic conditions at elevated temperatures [24,25]. Unfortunately, under these conditions, fermentation inhibitors are produced during pretreatment of biomass and include sugar degradation products such as furfural and 5-hydroxymethylfurfural (HMF), lignin degradation products such as phenolic acids, alcohols and ketones, plus acetate from deacetylation of hemicellulose. These inhibitors can affect the fermentation microorganism's ability both to produce ethanol and to grow, depending upon the type of ethanologen selected [26]. The concentrations of the inhibitors present after pretreatment varies depending upon the pretreatment technology and fermentation feedstock, and concentration as low as 5 mM of any inhibitor can impact the fermentation, depending on the ethanologen used [27]. Existing technologies that have been investigated for inhibitor removal include ion exchange and membrane-based technologies [28], polymeric adsorbent [29], chemical agents that precipitate contaminants such as Ca(OH)_2 _– often referred to as overliming [30] – and solvent extraction [31]. Unfortunately, all these approaches are only partially effective, add considerable costs to the fermentation process, and still leave much of the various inhibitors in the process streams. Any attempt to recycle and reuse process water is significantly limited due to build-up of these inhibitors, even though water recycle was reported to be a critically important parameter in biorefinery process integration especially with the industrial requirement for high solids loading of > 20% w/w that yield higher concentrations of inhibitors [32].

The use of MFCs for removal of acetate has been reported previously [33-35]. Here, we demonstrate the removal of the fermentation inhibitors produced during biomass pretreatment including the sugar degradation products (furfural, HMF) and lignin degradation products (phenolic acids, aldehydes and ketones) with simultaneous electricity production. This is the first study investigating electrogenic conversion of furans and phenolic molecules. One study reported the effect of the inhibitors on sugar conversion in MFCs, but not the transformation of these molecules themselves [36]. MFC parameters such as power density, coulombic efficiency, composition of the anode microbial community and stability were investigated. The impact of including an MFC in the biorefinery process recycle stream for inhibitor removal is discussed. A number of factors including inhibitor levels, degree of mineralization, byproduct formation, MFC performance, stability, maintenance, contamination, cost, effect on ethanol yield and others were considered in determining the suitability of applying MFCs for controlling inhibitor concentration in biorefinery recycle streams and consequentially improving the potential for water recycle and reuse.

## Results

### Enrichment of microbial consortium

**Figure 2:**
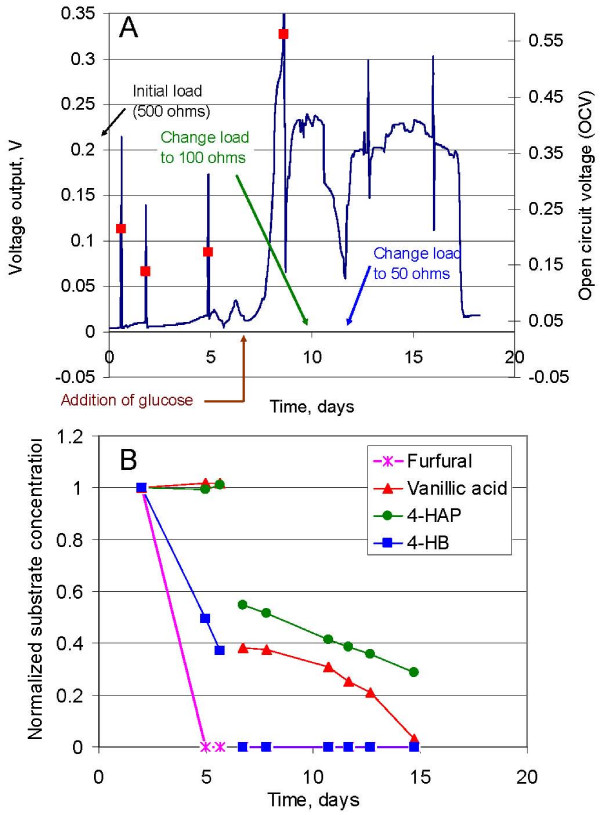
**Removal of fermentation inhibitors with simultaneous production of electricity**. The voltage output during the growth of the microbial consortium in MFC-A is shown in section A. The open circuit voltage (red squares) is plotted on the secondary Y axis. The removal of fermentation inhibitors used as substrates in the MFC follows the following trend: 2-furfural, HB, Vanillic acid, 4-HAP (Section B).

The inoculum obtained from an acetate-fed MFC (Borole, et.al., Improving power production from acetate-fed microbial fuel cells via enrichment of exoelectrogenic organisms in continuous flow systems, unpublished manuscript) was enriched on a mixture of fermentation inhibitors including 2-furfural, 4-hydroxybenzaldehyde (HB, model phenolic aldehyde), 4-hydroxyacetophenone (HAP, model phenolic ketone) and vanillic acid (VA, model phenolic acid). Growth of a microbial consortium using these molecules as the carbon and energy source was studied. Current production in MFCs is typically assessed by applying an external resistance across the anode and cathode (closed circuit) and measuring the voltage output. During startup, the carbon source is typically used for microbial growth which results in an increase in the voltage output [37]. Change in voltage in a closed circuit is typically used to measure the amount of electricity that can be produced. Figure 2A shows the increase in voltage output from the MFC with an applied external resistance of 500Ω. The spikes in the voltage curve indicate open circuit voltage (OCV) measurements. The OCV increased to 0.56 V in the first 9 days. The voltage output (in closed circuit) during this time was low, indicating that part of the carbon source was being used for growth of microorganisms. The total electron recovery in the form of current during the 17-day experiment was about 7%. A specific order of depletion was observed for the model compounds studied, as indicated via monitoring of the substrate molecules by high performance liquid chromatography (HPLC) in real time. 2-furfural was the first one to be consumed, followed by HB, VA and HAP, in that order (Figure 2B). The trend in the voltage output (under closed circuit conditions) correlated with the consumption of the individual substrates, For example, the initial voltage peak of 0.017 V on day 5 corresponded with depletion of 2-furfural, followed by a peak of 0.03 V on day 7 corresponding with the depletion of HB. The concentration of the inhibitors remaining after day 7 was reduced by dilution with fresh medium at a ratio of 1:3, to minimize potential inhibitory effects of the remaining phenolic compounds and to promote further growth. Glucose was also added on day 7 to promote microbial growth and was consumed immediately with a concomitant rise in voltage output to 0.3 V (day 8) as shown in Figure 2A. The consumption of VA was observed between day 9 and day 15. Consumption of HAP was initiated on day 7, and continued beyond day 15. The voltage output decreased to below 0.02 V on day 18, indicating lack of substrate availability. A relatively stable voltage was obtained between day 13 and day 18 (0.22 ± 0.02 V). The MFC was operated at a 50Ω load thereafter and a voltage of 0.15 V or higher was obtained when adequate substrate was provided. The microbial consortium was investigated with a potential biorefinery application in mind; as such, the ability of the consortium to handle a broad substrate range, streams with multiple substrates present at the same time and concentrations representative of biorefinery streams was examined. After the initial enrichment, studies with individual substrates as well as multi-substrate studies were carried out, including HMF to broaden the substrate range further.

### Effect of the inhibitor concentration

The concentration of fermentation inhibitors in a pretreated biomass slurry or a typical biorefinery recycle stream ranges from a few mM to 20 mM or more [23,27]. The effect of the fermentation inhibitors on ethanol production in a cellulosic fermentation process increases with the concentration [27]. The effect of 2-furfural on electricity production was studied at a concentration of 0.1 g/l to 2 g/l (20.8 mM). Electricity production was observed at all concentrations without any decrease in the voltage output (under closed circuit) with increasing concentration. The voltage output did not increase either, with increasing substrate concentration, since the substrate was added in a fed-batch manner and even at a low concentration of 0.1 g/l the concentration was sufficient to produce steady voltage for a few hours. Under these conditions, the maximum voltage output was limited by the cathodic reaction, as indicated by operation of the MFC with a ferricyanide cathode, which showed much higher current output. Cathode limitations have been demonstrated previously [11,38,39] and also for the MFC configuration used in this study (unpublished study). Electricity production was also observed with the other substrates at all concentrations, but the voltage output was lower. The concentrations of other substrates examined were as follows: HMF (0.1 to 2 g/l), HB (0.1 to 1 g/l) and VA (0.1 to 4 g/l).

**Figure 3:**
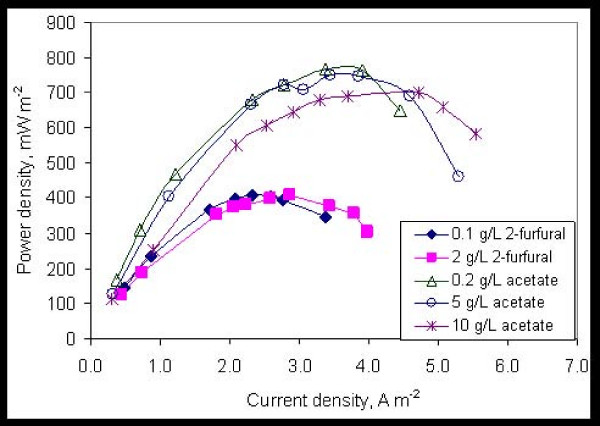
**Removal of 2-furfural and acetate from an aqueous stream with concentrations up to 2 g/l and 10 g/l, respectively, demonstrates tolerance of the MFC consortia to high concentrations of inhibitors**. The power density of the MFC remains constant above a threshold inhibitor loading since the power output is limited by the air cathode, vs the substrate concentration on the anode side.

The effect of acetate concentration on electricity production was also studied in a different MFC (MFC-B) using the consortium enriched on acetate. The power density obtained using acetate and 2-furfural as substrates in two different air-cathode MFCs at various concentrations is shown in Figure 3. The concentration of the fermentation inhibitor did not have any adverse effect on the power density, but instead resulted in an increased current density. The higher current densities at the high concentration indicate a high level of substrate tolerance by the microbial consortium.

### Substrate conversion and coulombic efficiency

The degree of conversion of the inhibitor molecules is an important parameter during consideration of the MFCs for application in biorefinery recycle water clean-up. HPLC analysis of the samples during the course of the run showed the appearance of a few intermediates which were eventually consumed. The intermediates were not characterized in this study, except to determine the retention time by HPLC. The removal of 2-furfural and HMF was near complete. The coulombic efficiency (CE) of an MFC is the ratio of the total charge recovered from the MFC as electricity to the maximum charge possible from complete conversion of the substrate to electricity. The CE for furfural, HB and HMF was 69 ± 3%, 64 ± 4% and 60 ± 4%, respectively. The CE for conversion of VA and HAP was much lower due to low voltage output from these two substrates, and therefore was not quantified. Although the voltage output from the MFC using VA and HAP was low, these substrates were removed by the microbial consortium in the MFC.

### Maximum power density of the MFC

**Figure 4:**
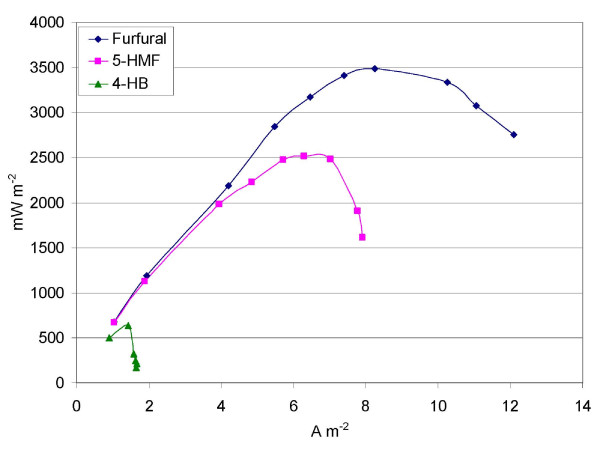
**Power density curves for fermentation inhibitor removal in MFCs at a concentration of 0.2 g/l, using a ferricyanide-cathode**.

Power density is an important parameter for determining the cost-effectiveness of MFCs if electricity production is the sole purpose of their application. Water recycle and ethanol yield are more important parameters in the case of the biorefinery application. Nevertheless, energy recovery from the process has to be considered in the overall economic feasibility. The use of a ferricyanide-based cathode has been shown to result in higher power densities as compared with a Pt-based air-cathode in MFC studies [37], and its use allows determination of the electron production ability of the anode biocatalyst. Each of the inhibitors was studied individually with the ferricyanide cathode. The results for 2-furfural, 5-HMF and HB are shown in Figure 4. A maximum power density of 3490 mW/m^2 ^(336 W/m^3^) was obtained for 2-furfural as the substrate. HMF produced 2510 mW/m^2 ^(238 w/m^3^), while HB produced 630 mW/m^2 ^(62 W/m^3^).

The electricity produced with VA and HAP as substrates was 150 mW/m^2 ^and 9 mW/m^2^, respectively (data not shown). It was observed that the power output from the MFC using these phenolic substrates increased with time. It should be noted that the power density analyses with the phenolic substrates was conducted post-operation of the MFC with furan substrates (2-furfural and HMF) for a prolonged period (45 days). Since a substrate preference was also observed during initial enrichment (Figure 2), it is likely that the microbial population shifted towards organisms that would use the furan molecules as substrates. A low population of microbes capable of using HB, VA and HAP as substrates in the consortium can potentially explain the low power density observed for these substrates.

### MFC stability

The MFC was operated for a total period of 10 months. It was operated with an air cathode for the first 6 months, and with a ferricyanide cathode, thereafter. The operation was conducted in a closed-loop anode liquid recirculation mode with an intermittent change of the liquid medium as indicated in the Methods section. The voltage output of the MFC was dependent on the substrate used. Using 2-furfural as the substrate, the voltage output was relatively stable (at 0.15 ± 0.02 V at a 50Ω load), if adequate substrate was provided and the anode pH was maintained. A pH polarization was observed in the MFC as reported elsewhere for two-chamber MFCs [40]. The stability of the system was assessed during unplanned events of substrate limitation and pH drop. After operation of the MFC without substrate for a 24 to 48 hour period, the voltage output was found to return to 0.15 ± 0.02 V within 1 to 2 hours of substrate addition. A similar observation has been reported in other MFC studies investigating short-term substrate starvation [41]. Similarly, after operation of the MFC at a pH below 6.0 for a period of 24 to 48 hours, a similar response was observed. The MFC was operated with a ferricyanide cathode for a 4-month period, during which the high power density was maintained and actually increased up to 3700 mW/m^2^, with 2-furfural as the substrate. This indicated the relative stability of the power output and the MFC operation.

### Microbial characterization

**Figure 5:**
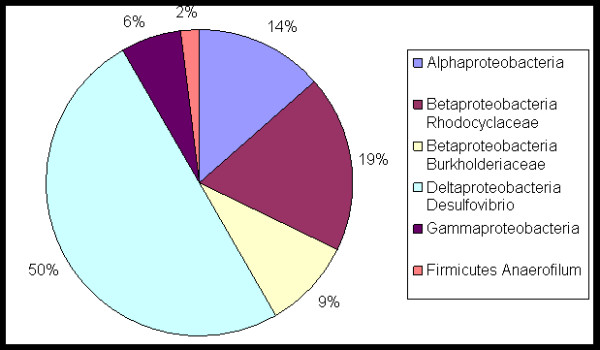
**Distribution of microbial population in MFC enriched on fermentation inhibitors to the Class level or below**.

A total of 95 clones were sequenced from a library created from the sample collected on day 83 from the MFC anode Clone library, Accession # FJ823862 – FJ823945). Eleven sequences were excluded from phylogenetic analyses because they were found to be chimeras. The microbial community was dominated by δ-Proteobacteria which constituted 50% of the clone library population (43 clones from a total of 84 clones) (Figure 5). The closest match to this group was a previously uncultured environmental bacterium *Proteobacterium *Core-3 enriched in a consortium in an electrochemically-assisted bioreactor using iron for respiration. The 43 strains were not all similar and exhibited between 92 to 99% similarity to the strain Core-3. Additionally, the closest known genus similar to this group of organisms was *Desulfovibrio *(92 to 99% similarity). This group was designated as Group A (Table 1). The similarity data given here is based on 16S rRNA obtained using PLAN or BLAST [42].

**Table 1:** Distribution of microbial population in MFC.

*Clone #*	*Closest relatives of known Genus*	*Accession #*	*Query Coverage, %^†^*	*% similarity*	*Group #^‡^*
A01, A04, A07, A09, A10, A11, A12, B01, B03, B08, B09, B10, B11, C03, C04, C05, C07, C10, C11, C12, D01, D02, D03, D06, D10, E03, E05, E07, E11, E12, F03, F05, F08, F11, F12, G01, G04, G10, G11, H02, H04, H07, H10	*Desulfovibrio intestinalis*, strain KMS2	Y12254.1	98	92–99	A

B12, C01, D07, E09, F01, F04, F10, G02, G09, H01, H03, E10	*Azospira oryzae *strain N1	DQ863512.1	100	89–97	B

B04, B05, D11	*Azospira oryzae *strain N1	DQ863512.1	100	94–95	C

A08, B06, B07, C06, C09, D05, D08, E02, G03	Proteobacterium LS-1	AB111107	92	95	D

D09	*Anaerofilum agile*	X98011.1	97	89	

C08, F06	*Dysgonomonas gadei *strain 1145589	Y18530.1	98	95	

C02	Fenthion-degrading bacterium FP1–6	DQ120938.1	100	91–98	

A02	*Oscillibacter valericigenes *sp.	AB238598.1	97	95	

H05, E01	*Ralstonia eutropha *H16	AM260479.1	100	92	

G05	*Ralstonia metallidurans *CH34	CP000353.1	100	98	

D12, F07, G07, H08	*Comamonas *sp. XJ-L67	EU817492.1	99	99	

A03, B02	*Ochrobactrum *sp. 1605	DQ989292.1	100	99	

E06	*Pseudomonas *sp. FP1–1	DQ118951.1	99	99	

G08	*Ralstonia *sp. PHD-11	DQ374436.1	100	90	

F02	Bacterium 7B9	DQ298776.1	94–100	90–94	

^† ^The query coverage and % similarity are based on 16S rRNA homology search conducted using nucleotide BLAST.

^‡ ^The group # is provided only for clones which were similar to potential exoelectrogens.

The closest known genus to the clone is listed. The group # refers to clones which were found to be similar to potential exoelectrogens reported in literature.

**Figure 6:**
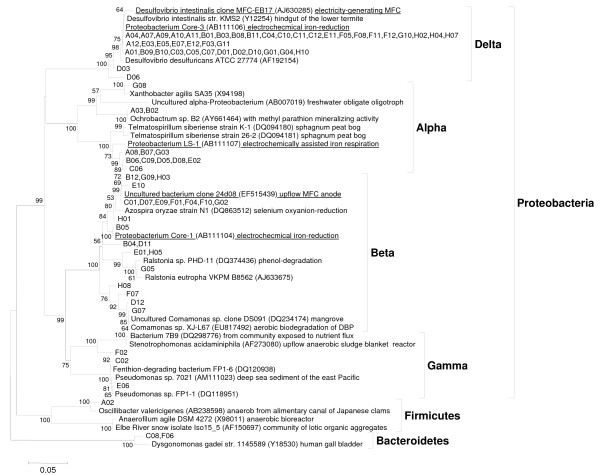
**Phylogenetic tree of MFC anode consortium enriched using fermentation inhibitors as the carbon and energy source**.

The remaining 41 clones in the library had significant diversity, representing more than 14 genera. Of these 41 clones, 24 clones were genetically similar to potential exoelectrogens reported in the literature. These 24 clones were divided into three groups based on phylogeny (B to D, Table 1). Group B (12 clones) showed 94 to 99% similarity to a strain 24d08 (EF515439) enriched in an MFC by Angenent's laboratory This group was similar to the genus *Azospira *(89 to 97% similarity). Group C (3 clones) exhibited 95% similarity to a *Proteobacterium *Core-1, which came from the same electrochemical bioreactor as strain Core-3. Group D (9 clones) exhibited 94 to 97% similarity to an organism *Proteobacterium *LS-1, which was also from the same electrochemical bioreactor as strain Core-3. The phylogenetic tree for the MFC community is shown in Figure 6. Thus, 80% of the population showed some level of phylogenetic similarity to potential exoelectrogens reported in literature. The details of the percent similarity and query coverage for all clones are available in the Table 1. The other clones demonstrated similarity to organisms capable of degradation of various xenobiotic molecules including phenols, methyl parathion and fenthion, as well as those capable of selenium reduction. Some of these organisms may also be exoelectrogenic; however, the closest organism to these clones based on similarity analysis using BLAST was not a known exoelectrogen.

## Discussion

A proof of principle for removal of fermentation inhibitors from aqueous streams using MFCs is demonstrated. In order to assess the potential applicability of the MFCs for removal of fermentation inhibitors from biorefinery process streams, the following points are considered: 1) percent removal of fermentation inhibitors and degree of mineralization; 2) ability to handle high concentrations of inhibitors, representative of biorefinery streams; 3) performance of MFCs with mixed-substrate feed (multiple inhibitors at the same time); 4) stability of the MFC performance including power output and identification of parameters requiring control; and 5) total power generated by implementing MFCs in biorefineries.

### MFC performance

The removal of six model fermentation inhibitor compounds via electrogenic conversion was studied in this work. Near-complete removal of 2-furfural and HMF was observed, although it required continuous recirculation of the aqueous phase. Further work with continuous flow and single-pass operation is needed to further improve the efficiency of operation. Removal of 2-furfural was observed with a final concentration (including all byproducts) below 0.05 mM (99% removal) at the laboratory scale. The CE data indicated 60 to 69% conversion of the inhibitors to electricity. The balance of 31 to 40% of the inhibitor was probably assimilated into cells or biofilm since no persistent byproducts were observed. Experiments with inhibitor concentration up to 20 mM did not prevent electricity production, indicating that the model compounds tested were not inhibitory to the anodic microbial biofilm consortium. The ability to handle multi-substrate mixtures containing 2-furfural, HB, VA and HAP was also demonstrated. The effects of byproducts produced during MFC treatment on fermentation are important criteria in determining the success of this technology in its application to the biorefinery process. The HPLC data indicated near-complete mineralization of the 2-furfural and HMF, thus implying minimal potential for any adverse effects on the fermentation process. Further work is needed to investigate the byproducts from the lignin degradation model compounds.

The long-term stability of the MFC depends on implementing process control to maintain key operating parameters in a desired range. Some of these parameters include pH, substrate loading, flow rate and oxygen transport into the anode chamber. A pilot-scale MFC study identified a number of parameters including these that need to be controlled to enable stable MFC operation [43]. The operational stability of the MFC with respect to power output was investigated and was found to be quite stable, provided sufficient substrate and neutral pH were maintained. After the initial inoculation, the anode biocatalyst did not require any replacement and functioned stably with nutrient replacement, substrate addition, pH control, and periodic flushing of the anode to remove planktonic cells, or excess microbial biomass over the complete 10-month period.

### Microbial diversity in exoelectrogenic consortium

This is the first report on direct conversion of sugar and lignin degradation products into electricity. Therefore, the microorganisms involved in the biochemical conversion are novel. None of the 84 clones sequenced showed 100% similarity to any known organism. Three of the 84 clones showed 99% similarity to identified organisms, of which one was a *Comamonas *sp. and the other two *Pseudomonas *spp. The potential exoelectrogens identified in the MFC-A demonstrated 89 to 99% similarity to reported exoelectrogens. Although about 80% of the clones sequenced from the 16S clone library of the MFC sample showed phylogenetic similarity to known exoelectrogens, very little is known about how the organisms may be interacting with electrode materials. The phylogenetic tree (Figure 6) demonstrates the significant microbial diversity of these organisms and indicates that such organisms may be present in diverse environments such as termite gut, anaerobic sludge reactors, sediments, metal-reducing environments, insecticide-contaminated environments, and so on.

The enrichment strategy used in this study targeted selection of biofilm-forming, exoelectrogenic organisms, reported previously with sugars and organic acids as the energy source [15]. A compact, flow-through anode design and a dual flow-rate regime was used to minimize non-biofilm forming organisms. The inoculum used for this enrichment was obtained from an acetate-fed MFC (Clone library Accession # FJ823774 – FJ823861). The dominant organisms in the acetate MFC were from the class β-Proteobacteria (71%), followed by δ-Proteobacteria (13%) and γ-Proteobacteria (5%). Enrichment with furan and phenolic molecules as substrates resulted in substantial change in the population resulting in a consortium containing 50% δ-Proteobacteria, 28% β-Proteobacteria and 14% α-Proteobacteria. The genus closest to the most dominant organism in the consortium enriched in this study was *Desulfovibrio *(98% coverage and 99% identity). Only one other study has reported species from this genus in MFCs [44]. The microbial consortium enriched in this study is unique in its ability to transform fermentation inhibitor into electricity. The consortium was enriched on a mixed substrate carbon source and operated with a synthetic mixture of fermentation inhibitors to assess the biocatalyst response, and as such, requires further investigation using real biorefinery streams. It is likely that MFC microorganisms will preferentially remove sugars prior to removal of the acetate or other inhibitors. While this may be the case with natural consortia, development of substrate-specific exoelectrogens that can only remove the inhibitors may also be possible.

### Implications for biorefinery process water recycle

**Figure 7:**
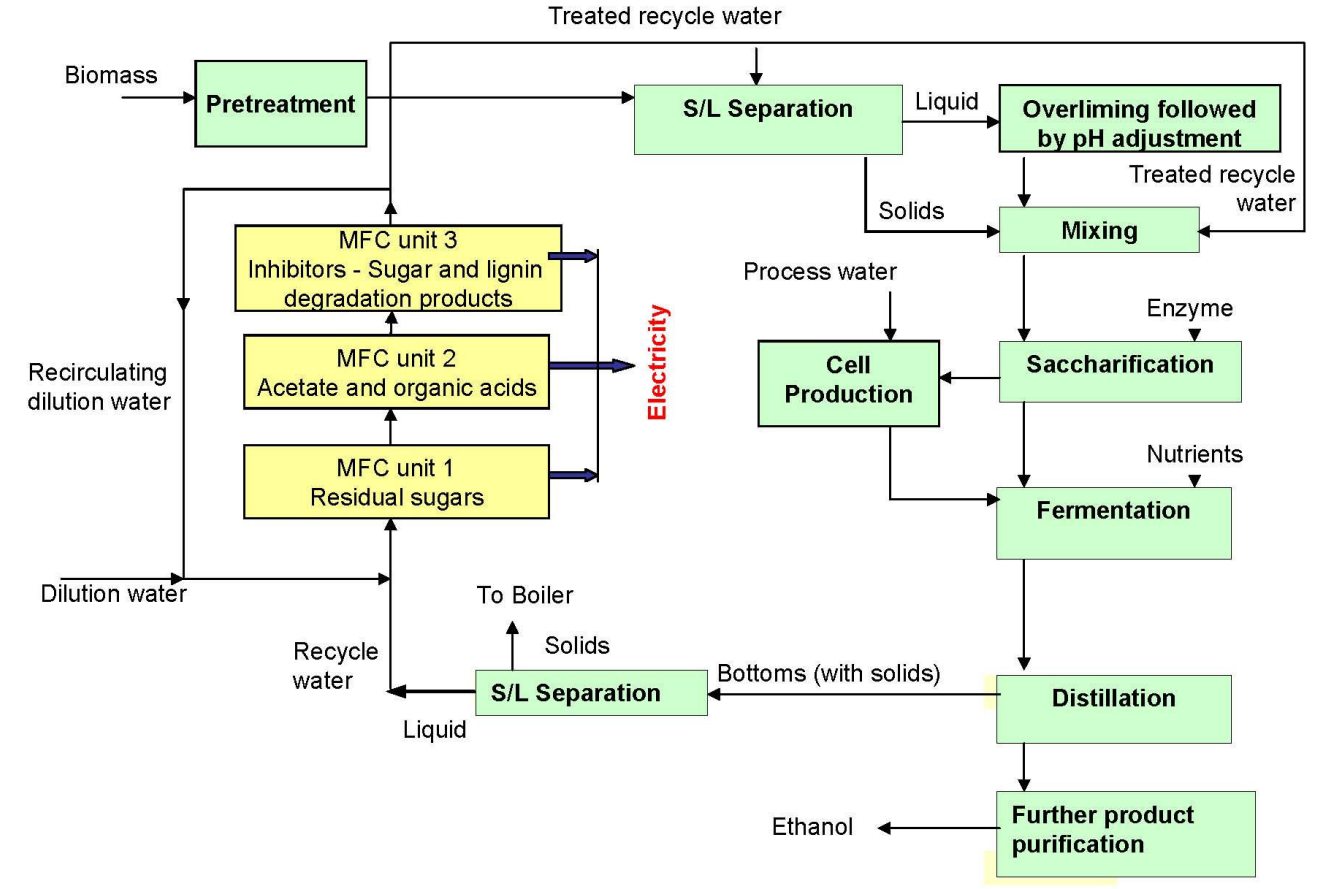
**Biorefinery flow sheet showing location of MFC units capable of removing specific substrates from the process recycle stream**. The flow sheet was based on conversion of corn stover to ethanol via dilute acid hydrolysis pretreatment process reported by NREL [32].

In a biorefinery process, a potential point of application for MFCs is downstream from the ethanol recovery unit (for example, distillation column) after the solid-liquid separation unit (Figure 7). The MFCs may be placed in the recycle stream returning to the saccharification/fermentation vessel via the mixing chamber. A once-through process is preferable; however, a recirculation within the MFC unit operation is most likely necessary to achieve complete removal of the inhibitors.

The amount of electricity produced from the bioconversion of the inhibitor molecules is a function of the CE, power density and the concentration of the inhibitor molecules in the recycle stream. Figure 7 shows the overall process schematic including MFCs which is based on a flowsheet model from the National Renewable Energy Laboratory (NREL) for biochemical conversion of corn stover to ethanol via a dilute acid pretreatment method [23,32]. This process was considered for determination of potential power output from the MFC system. The concentrations of the inhibitors in the recycle stream were obtained from the NREL report [23]. This process assumes a solids loading of 30%. Acetic acid, 2-furfural, HMF and glucose and its oligomers are present in the recycle stream at a concentration of 6.5, 1.5, 0.23 and 2.1 g/l, respectively. Since it was observed that glucose was consumed prior to the removal of the inhibitor molecules by the MFC consortium, it was also considered a substrate in the MFC. A minimum of three different MFCs were presumed to be needed, each with a certain substrate specificity (namely, sugars, acetate or organic acids, and lignin- + sugar degradation products). Assuming a 60% CE, the process yielded 2.5 megawatts of power. This is equivalent to one quarter of the total power needed for the biorefinery plant. The MFC technology is presently limited by various losses that occur during energy harvesting at higher scales due to electrochemical and process issues. These limitations can reduce the overall power that can be harvested from the biorefinery process water. Production of hydrogen instead of electricity has the potential to result in a reasonable economic advantage for the overall biorefinery process.

MFC technology is in its early stage of development. A detailed economic analysis of MFCs has not been reported to date. Preliminary analyses suggest a minimum power density of 0.5 to 1 kW/m^3 ^to consider commercial application [3,4]. Recent studies on the MFC anode design and control of operational parameters have resulted in developments which have improved the application potential [3,9,11,15,43,45,46]. Use of alternate membranes [47] and sustainable cathodes [8,16-18] has revealed potential alternatives for other MFC components. Process control strategies optimizing substrate loading, flow rates, pH, ionic strength and dissolved oxygen levels in the feed have potential to improve the operational feasibility of an MFC-based process.

A number of other factors including operational feasibility of MFCs, (lifespan of cathode and membrane, maintenance requirements during periodic and/or annual shut-downs, etc.), quality of recycle water after MFC treatment (presence of nuisance dissolved and suspended solids), effect of using MFCs on ethanol yield from biomass, and finally, overall effect on the economics of the biorefinery process, also need to be considered.

A biofilm-based process such as that developed in this work would enable flow of the recycle water through the MFC anode to remove the fermentation inhibitors, with relatively clean water exiting the MFC system. A minimal separation step may be needed, if any, since the aqueous stream would not contain a significant amount of microorganisms, especially if a periodic wash step is employed in a separate flow loop to remove excess biofilm and planktonic cells. The most important impact the MFC can have on the biorefinery process is the potential indirect effect on the ethanol yields by limiting accumulation of fermentation inhibitors. NREL's analysis has shown that for a solids loading of 25%, increase of water recycle from 10% to 25% results in a reduction in the ethanol yield from 65% to 5%, potentially due to the accumulation of the inhibitors [32]. The acetate concentration may be considered as a representative for all inhibitors present in the hydrolyzate, some of which may have more detrimental effect on the ethanol yield. The need for recycling of water in a biorefinery and in the biofuels industry in general has been stressed recently [48,49] with the conclusion that the industry might be limited in size and/or location based upon water availability.

The feasibility of MFC application in a biorefinery requires a demonstration of economic advantage of such a scheme over the existing process scheme. The power produced from conversion of the fermentation inhibitors to electricity is only a side benefit compared with the positive economic impact of removal of the fermentation toxins. Further investigation is required to fully understand the potential for fermentation inhibitor removal by MFCs, especially regarding the potential of further microbial consortium evolution toward improved efficiency. Increased MFC efficiency impacts the degree of process water recycle possible. This latter aspect potentially improves both the production plant's economics, and may broaden environmentally acceptable green-field plant sites available due to reduced groundwater requirements.

## Methods

### MFC construction

The MFC used in this study was a two-chamber design (Figure 1). The microbial enrichment was carried out using an air cathode, consisting of a platinum-deposited carbon electrode (Fuel Cell Store, # GDE HT 140 W-E). The anode chamber (4 cm diameter × 1.27 cm thickness) contained a carbon felt (Alfa Aesar, part # 42107) electrode separated from the cathode by a Nafion-115 membrane. The anode chamber was a compact, flow-through system with high electrode surface area to volume ratio (45,230 m^2^/m^3^). The projected surface area of the anode was 12.57 cm^2 ^and was used to calculate power density. Additional details of the MFC design are reported elsewhere [15]. A ferricyanide-cathode was used for determining the maximum power density.

### Inoculation and operation

The anode chamber of the MFC (MFC-A) was inoculated with a 10 ml culture sample from an MFC enriched with acetate as the energy source, which was enriched from an anaerobic digester sample (Borole, et.al., Improving power production from acetate-fed microbial fuel cells via enrichment of exoelectrogenic organisms in continuous flow systems, unpublished manuscript). The inoculum sample was obtained by dislodging the biofilm from the anode surface of the acetate-fed MFC with a hypodermic needle, followed by withdrawal of the sample with a syringe. The inoculum was added directly into the flow line entering the anode chamber and carried into the anode chamber by the de-aerated recirculating medium. The nutrient medium was prepared as described previously [15] and was placed in a glass bottle reservoir (anode liquid reservoir, 200 ml) and recirculated through the anode chamber at 4 to 7 ml/min. The medium was de-aerated by bubbling nitrogen. The enrichment process included replacement of the recirculating medium intermittently (whenever optical density at 600 nm increased above 0.05) to minimize planktonic bacteria and mediators and enrich exoelectrogenic biofilm-forming organisms [15].

The carbon source added to the anode liquid reservoir was a mixture of fermentation inhibitors. It included 0.2 g/l 2-furfural, 0.1 g/l HB, 0.1 g/l HAP and 0.5 g/l VA. Acetate was also added for the first seven days in a continuous manner using a syringe pump at the rate of 0.2 g/l per day. All other substrates were added all at once at time zero and tracked until complete disappearance. The anode medium was amended with 0.4 g/l glucose on day 7 primarily to enhance the rate of microbial growth. The initial external resistance (load) applied to the MFC-A was 500Ω, which was reduced to 100Ω when the voltage output increased above 0.2 volts (day 10) and then to 50Ω (day 12). The MFC was tested with individual inhibitors from day 20 to day 50, by adding them at 0.05 to 0.2 g/l. The conversion of HMF was also investigated subsequently. A second experiment was conducted with acetate using a different MFC (MFC-B) to examine the conversion of acetate. The details of the MFC-B and the consortium are reported elsewhere (Clone library Accession # FJ823774 – FJ823861).

### Effect of inhibitor concentration on the microbial consortia

After confirmation of electricity production from individual inhibitor molecules, the effect of inhibitor concentration on their removal and electricity production was studied. The experiments were conducted by adding each of the substrates individually, at various concentrations to study substrate inhibition. From day 51 to 62, HB was used as the energy source for the MFC at concentrations from 0.1 to 1 g/l. From day 63 to day 82, 2-furfural was used as the energy source at a concentration from 0.05 to 2 g/l. From day 83 to day 101, VA was used as the substrate from 0.05 to 4 g/l, followed by use of HMF as substrate from day 129 to day 150 at a concentration from 0.2 to 2 g/l. The MFC was operated with 2-furfural as the substrate between day 102 to 128. The effect of acetate was studied in MFC-B using a different consortium (Clone library Accession # FJ823774 – FJ823861) up to a concentration of 10 g/l. The voltage output was monitored at each concentration for a period of up to five days.

### Power density analysis

The power density analysis for the MFCs was conducted by adding the energy source (inhibitor) to the anode liquid reservoir. The nutrient medium was completely replaced prior to every analysis, followed by fed-batch addition of the substrate into the medium (at 0.2 g/l). Two different cathodes were used for the power density analysis: the air cathode and a ferricyanide-cathode. A 200 mM potassium ferricyanide in 100 mM potassium phosphate buffer was used as the catholyte for the latter experiment. The analysis was conducted 60 minutes after addition of the carbon source, to allow the voltage output to stabilize. A variable resistor ranging from 0 to 5000 ohms was used and the voltage recorded by a Fluke multimeter Model 83. The resistance sweep was conducted at an interval of 5 minutes. The maximum power density was confirmed by operating the MFC at the particular resistance for at least one hour, following the power density analysis. Multiple measurements of the voltage output at the resistance exhibiting maximum power density were made on different days to determine reproducibility of the power density curve. The results were found to be within a 10% standard deviation.

### Genetic characterization

*16S clone library*. Microbial samples were collected from the anode of MFC on day 83 by dislodging the cells from the electrode using a hypodermic needle, followed by withdrawal of the cells using a syringe from the exit of the MFC anode. Genomic DNA was isolated using the standard freeze-thaw procedure, followed by phenol-chloroform extraction [50]. The 16S rRNA analysis was done as reported previously [15]. Multiple sequences were initially aligned against the most similar sequences in the Ribosomal Database Project II (RDP II) and assigned to a set of hierarchical taxa using a Naïve Bayesian rRNA classifier version 1.0 . Orientation of the sequences was checked using program OrientationChecker v.1.0 available at . Sequences with unknown orientation were omitted from further analyses. Clone libraries were checked for the presence of chimeric sequences using a program Bellerophon [51] and the Chimera Detection program in the RDP II . Putative chimeras were excluded from further analyses. Closest relatives were retrieved from NCBI GeneBank following BLAST search [42]. To determine the clone library coverage for each sample, statistical analyses were performed using DOTUR [52]. The population distribution is reported as a percentage of the total number of bacterial clones sequenced for each sample.

## Conclusion

In this work, an MFC capable of reducing the concentration of known fermentation inhibitors including the sugar-degradation products, furfural and 5-hydroxymethylfurfural, and lignin degradation products, vanillic acid, 4-hydroxybenzaldehyde and 4-hydroxyacetophenone was demonstrated. Use of an integrated MFC design resulted in high power densities, reaching up to 3700 mW/m^2 ^(356 W/m^3 ^net anode volume) and a coulombic efficiency of 69%. A unique exoelectrogenic microbial consortium capable of broad substrate specificity, ability to handle high inhibitor concentrations (5 to 20 mM) with near complete removal, and stable long-term power generation was identified and characterized. The dominant organism in the consortium was found to be a δ-Proteobacteria, which was phylogenetically close to *Desulfovibrio *spp. The MFC-based approach for removal of fermentation inhibitors has implications for: 1) enabling higher ethanol yields at high biomass loading, 2) improved water recycle and 3) electricity production up to 25% of total cellulosic biorefinery power needs.

## Competing interests

A patent application related to MFC use in biorefineries is in preparation.

## Authors' contributions

APB made contributions to conception, design, analysis and interpretation of data and wrote the first draft of the manuscript; JRM was involved in data interpretation, manuscript preparation and revisions; TAV performed the 16S rRNA analysis and contributed to manuscript revision; and CYH contributed to experimental design and analysis. All authors read and approved the final manuscript.
